# A Novel and Simple Method for Laboratory Diagnosis of Relapsing Fever Borreliosis

**DOI:** 10.2174/1874285800802010010

**Published:** 2008-01-14

**Authors:** Christer Larsson, Sven Bergström

**Affiliations:** Umeå University, Department of Molecular Biology, Laboratory for Molecular Infection Medicine Sweden (MIMS) SE-901 87 Umeå, Sweden

**Keywords:** *Borrelia*, relapsing fever, diagnostics, detection, fever

## Abstract

Relapsing fever caused by *Borrelia* bacteria is often obscured by malaria and incorrectly treated. Here a novel method for diagnosis is presented. The method is cheap, simple and requires minimal laboratory material. Despite its simplicity, the method shows surprisingly high sensitivity, detecting concentrations less than 10 bacteria/ml blood.

## INTRODUCTION

Relapsing fever (RF) is a disease caused by several species of bacteria belonging to the genus* Borrelia*. The illness occurs worldwide in tropical and sub-tropical areas, but is most common in Africa [1, 2]. In some areas, the incidence is the highest for any bacterial disease on the continent [3]. All species pathogenic to man are transmitted by ticks of the genus *Ornithodoros* with the single exception of *B. recurrentis, *which is transmitted by lice [4, 5].

The main manifestation is, as the name indicates, a recurring fever with massive amounts of bacteria in the blood during fever peaks [1, 2, 6]. The relapsing pattern is due to the reciprocal action between host antibody response and bacterial antigenic variation of surface proteins [7, 8]. However, fever is a common symptom of many diseases and RF is often atypical or causes mild or sub-clinical disease. Therefore it is neglected and overlooked in many areas, often mistaken as malaria and hence incorrectly treated. In Togo, West Africa RF was previously unknown although it was recently found to be the causative agent in about 10% of the fever patients [9]. The mortality ranges from 2-40% and is highest in children. RF also cause pregnancy complications such as stillbirth [2].

During fever peaks, diagnosis is simple since there is massive bacteraemia easily detected with e.g. the Giemsa staining used in malaria diagnosis but between the peaks and in milder disease the bacteria are few and very hard to find in a stained blood smear by microscopy. Protocols used for malaria diagnostics in which water is used to lyse erythrocytes usually also lyse spirochetes and are therefore useless for RF diagnostics. Phase contrast or dark field microscopy directly on10-fold diluted blood is useful when spirochetemia is high. Due to the spirochetes’ thin and transparent morphology, ordinary light microscopy is very difficult and therefore of limited use.

The use of acridine orange–coated quantitative buffy coat tubes, centrifugation, and fluorescence microscopy is effec-tive [10, 11]. However, this technique requires expensive equipment seldom available in RF endemic areas.

PCR is an excellent method for RF diagnostics. Briefly, DNA is extracted from about 100 µl blood, analysed by PCR using *Borrelia*-specific primers and separation on an agarose gel, or if using real-time PCR, the results can be directly monitored on the computer screen in real time [12, 13]. The method is fairly quick and sensitive if the instrument and all reagents, including specific primers, are accessible. The major drawback with PCR is the cost of necessary equipment and reagents. It is also labour intensive and sensitive to contamination, which can result in false positive results. Smaller hospitals often have limited possibility of using PCR in their diagnostics.

ELISA is a valuable scientific tool to screen sera for antibodies against pathogens. Most useful is recombinant GlpQ, a protein found in all RF *Borrelia* but not in other related bacteria such as *B. burgdorferi *or *Treponema*. An ELISA detecting antibodies recognizing GlpQ identifies patients who have encountered RF and mounted an immune response against the pathogen [9, 14, 15]. RF is an acute disease and in most fatal cases, the first attack is most critical. This lasts for a few days and thereafter it takes another couple of days until the fever drops as a result of the host antibody response. It is not until this time point that there are sufficient antibodies for detection by ELISA. Although superior in identifying persistent low-grade disease, ELISA is expensive and labour intensive. It is more valuable for less acute diseases and for epidemiological studies of RF.

It is obvious that improved RF diagnostics is urgently needed. Therefore, we have developed this centrifugation-based enrichment method to provide a novel inexpensive and simple, but rapid and powerful RF diagnostic method to be used in rural health centres and perhaps also at larger hospitals.

## MATERIALS AND METHODS

### Setup of Diagnostics Protocol

Blood from healthy volunteers was drawn into heparinised VacuTainer tubes. *B. duttonii* was grown in BSK II culture medium [16] with 1,4% gelatin and 10% rabbit serum (Sigma) at 37°C, washed in phosphate buffered saline three times, counted in a Petroff-Hauser chamber using phase contrast microscopy. Bacteria were then spiked into 10 ml blood at known concentrations. The first centrifugation in which blood cells are separated from the plasma containing bacteria was optimized at a speed and duration to obtain a supernatant free from erythrocytes and leukocytes without lowering the concentration of bacteria. The second centrifugation in which bacteria are pelleted was optimized at a speed and duration at which no spirochetes remained in the plasma.

### Giemsa Staining and Microscopy

The plasma supernatant was aspirated and the almost invisible bacterial pellet was resuspended in the few remaining microliters of plasma. The suspension was smeared onto a glass slide as a “thick smear” the size of a coin and air-dried for a few minutes. The smear was then fixed by heating a few times over a flame followed by a 30 second dip in methanol. Staining was performed by a standard Giemsa staining protocol. Stained bacteria were visualized by light microscopy at 1000X magnification. Bacteria were either stained with Giemsa or directly visualized by phase contrast microscopy at 400X.

## RESULTS AND DISCUSSION

Although RF is one of the most common bacterial diseases in Africa it is often mistaken, and treated, as malaria, especially since they often occur as co-infections [17-19]. If effective treatment is given, this is often started only after a failed malaria treatment. After finding a 10% incidence of RF in fever patients seeking healthcare in Togo, West Africa, although the disease was unknown in the country, we realized that the need for improved RF diagnostics is urgent [9].

Methods such as the quantitative buffy coat technique using acridine orange and fluorescent microscopy, PCR with RF specific primers and ELISA for e.g. detection of antibodies to RF GlpQ protein are useful in diagnostics [10-15]. However, such techniques are often inaccessible, too lengthy or too expensive to be used where resources are limited.

**Fig. (1).Centrifugation-based enrichment method for spirochetes in blood.**
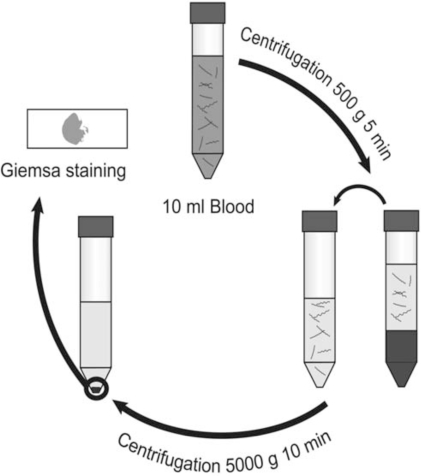
Venous blood is drawn into anticoagulant. Blood is thereafter centrifuged at 500 x g for 5 minutes, pelleting blood cells with most spirochetes remaining in the plasma. After transfer to a new test tube, the plasma is centrifuged at 5000 x g for 10 minutes to pellet bacteria. The supernatant is decanted, leaving spirochetes in the pellet. The suspended pellet is smeared onto a glass slide and stained with Giemsa for microscopic examination at 1000X magnification.

Therefore we have developed this centrifugation-based method to concentrate spirochetes from blood that can be used with minimal laboratory equipment (Fig. **1**). Methods involving ready-made PCR kits, density gradient centrifugation and Western blot detection of *Borrelia* proteins were initially considered. When we changed perspective and instead focused on the equipment and protocols already available at small hospital laboratories in RF endemic areas, we came to the conclusion that this approach is superior in its simplicity, rapidity, low cost and high sensitivity. The choice of Giemsa staining as a detection method makes it possible to process malaria and RF samples simultaneously since this is the stain most widely used in malaria diagnostics. A light microscope and Giemsa stain are the very basic equipment available at rural health clinic laboratories in malaria endemic areas.

**Fig. (2).Borrelia spirochetes concentrated from 10 ml blood and Giemsa stained.**
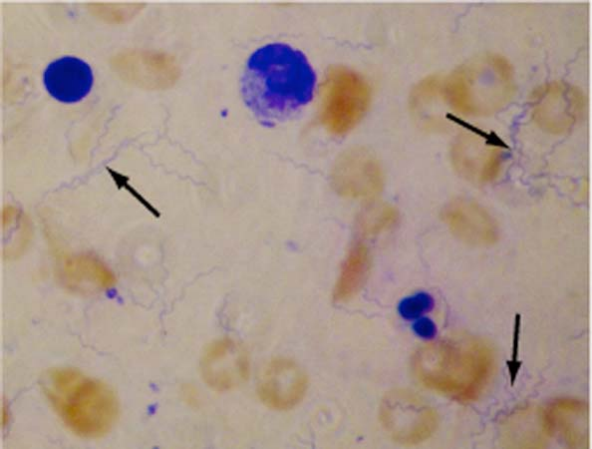
Low numbers of bacteria were concentrated as described in the material and methods section, Giemsa stained and visualized by light microscopy at 1000X magnification. Cells stained red and blue are contaminating erythrocytes and leukocytes, respectively. Some of the bacteria are marked by arrows.

The availability and quality of centrifuges differ tremendously. The first, slow centrifugation can be performed in any centrifuge. This was optimized to obtain a supernatant free from erythrocytes with minimal loss of bacteria. Since about 1/3 of the blood volume consists of erythrocytes a lot of bacteria is lost at this stage anyway. A 5-minute centrifugation at 500 x g was found to be optimal. Naturally, a slower/shorter centrifugation will give erythrocyte contamination in the second pellet which will conceal the thin, blue bacteria although small amount of erythrocytes is acceptable (Fig. **2**). At a faster/longer centrifugation the concentration of spirochetes in the supernatant gradually decreases.

The plasma supernatant was carefully transferred into a new centrifuge tube avoiding erythrocyte contamination from the pellet. The second centrifugation was designed to pellet all bacteria in the remaining plasma. A quite fast centrifugation is necessary to completely pellet all the bacteria. We found 5000 x g for 10 minutes to be sufficient but the time and speed can be extended without negative side effects. If the centrifuge is slower than 5000 x g, the time can be extended to maximize recovery although slower centrifugation often leaves some bacteria remaining in the supernatant which may lower the detection limit. Nevertheless, the sensitivity will be dramatically higher compared to direct microscopy of blood or plasma.

To evaluate the sensitivity of the method, 100 bacteria were spiked into 10 ml blood. The samples were processed as described and about 10 spirochetes were recovered on the glass slides by microscopy. This is a concentration of 10 bacteria/ml and according to our result the laboratory technician has a fair chance of diagnosing spirochetemia at a bacterial concentration of 1 bacteria/ml. This sensitivity may even be superior to many PCR-based protocols where the maximal volume blood to be used for DNA purification is about 100 µl.

If using a regular single-step PCR followed by separation with agarose gel electrophoresis and ethidium bromide staining, it takes approximately 500-1000 cells to get a detectable band (personal communication, J. Bunikis). The sensitivity can be increased by using nested PCR in which a second PCR is performed with two other primers, using the first reaction as template [9]. Nested PCR can detect as few as 1-10 bacteria but is very sensitive to contamination, resulting in false positive results. The sensitivity of real-time PCR, in which either incorporation of a fluorescent dye or DNA probe is measured, is similar to that of nested PCR [20]. By combining the methods into nested real time PCR, where an ordinary PCR reaction is followed by a real-time PCR with a probe and a new set of primers, the sensitivity is as low as 0.1-1 cell (personal communication, J. Bunikis). The small sample volume, typically about 100µl, used for DNA preparation may be more limiting than the sensitivity of the PCR methods when spirochetemia is low. By combining the centrifugation protocol with PCR, the sensitivity of RF detection can be further increased.

Due to its low-tech approach, mainly using equipment already available for malaria diagnostics and well-known procedures, we believe this novel technique will be very valuable for RF diagnostics, especially at rural health centres and hospitals. Due to the superior sensitivity it may also be considered to be the first-hand choice at larger hospitals, when suitable, in combination with PCR.
